# Anaesthesia for ophthalmologic surgical procedures in a patient with advanced amyotrophic lateral sclerosis: a case report

**DOI:** 10.1186/1757-1626-1-338

**Published:** 2008-11-20

**Authors:** Maciej M Kowalik, Tomasz Smiatacz, Robert Pajuro, Roman Skowroński, Hanna Trocha, Walenty Nyka, Krystyna Raczyńska, Maria Wujtewicz

**Affiliations:** 1 Department of Cardiac Anaesthesiology, Medical University of Gdañsk, ul. Dêbinki 7, 80-211 Gdañsk, Poland; 2 Department of Infectious Diseases, Medical University of Gdañsk, ul. Smoluchowskiego 18, 80-214 Gdañsk, Poland; 3 Department of Anaesthesiology and Intensive Therapy, Medical University of Gdañsk, ul. Dêbinki 7, 80-211 Gdañsk, Poland; 4 Department of Neurology, Medical University of Gdañsk, ul. Dêbinki 7, 80-211 Gdañsk, Poland; 5 Department of Ophthalmology, Medical University of Gdañsk, ul. Dêbinki 7, 80-211 Gdañsk, Poland

## Abstract

**Introduction:**

Anaesthesia procedures for surgical interventions in patients with amyotrophic lateral sclerosis (ALS) are not commonly found in clinical practice, and often have special considerations that must be taken into account in treatment planning. As a result, these procedures are rarely subject to publication, rendering it difficult for the anaesthesiologists to find useful and reliable information on this topic. ALS also presents a contraindication to the use of nondepolarising neuromuscular blocking drugs during general anaesthesia.

**Case presentation:**

In the case presented here, a 52-year old, White man, the progression of the disease to tetraparesis and respiratory failure, in addition to having the patient on chronic mechanical ventilation support, provided additional challenges to the handling team. The maturation of cataracts severely impaired communication with the patient, and surgical treatment of the cataract presented the only means to save communication. Such interventions are generally performed under local anaesthesia with the advice of the attending anaesthesiologist. However, in this case the patients' announcements during the operation would be unreadable to the advising anaesthesiologist. Here, the authors share experiences from a successfully applied combination of topical and general anaesthesia for two cataract operations and a vitrectomy. This was tolerated well by the patient, and without any side-effects.

**Conclusion:**

The applied treatment resulted in a substantial improvement of the vision and allowed communication to be maintained with the patient.

## Introduction

Anaesthesia procedures in patients with amyotrophic lateral sclerosis (ALS, Lou Gehrig's disease) often require certain special considerations [1]. However, surgical interventions in patients with advanced ALS are uncommon and, consequently, are rarely subject to publication, resulting in a lack of literature on this topic. To our knowledge there have been no reports published on anaesthesia procedures in ophthalmologic operations in patients with ALS. Thus, this work presents an example of successful anaesthesia procedures provided for two cataract operations in a man with advanced ALS on mechanical ventilation.

## Case presentation

A 52-year old, White man (height of 1.72 m, bodyweight of 78 kg) showed signs of limping in the right leg beginning in January 1998. No neurologic disorders had been previously diagnosed in the family. The patient had experienced a neck and occiput injury (struck with a stone) about 20 years earlier, but this was resolved without complications. Information on smoking was unavailable, and the patient occasionally consumed small amounts of alcohol.

Initially, the Neurology Department suspected a motor neuron disease and, due to initial uncertainties with the diagnosis, the patient was consulted at a national reference centre for motor neuron disease, where the diagnosis of ALS was ultimately confirmed 15 months after disease onset. Riluzole (Rilutek, Aventis Pharma, France) was introduced as the sole medication.

Twelve months after the disease onset the patient was forced to begin using a wheel chair, and, after another eight months, hypoventilation developed. The patient became dyspneic and was directed to the Infectious Diseases Department Intensive Care Unit to consider the option of chronic mechanical ventilation support. Following admission to the hospital, the patient was extensively informed about the benefits and disadvantages of chronic mechanical ventilation support, and a written consent was obtained. Tracheal intubation was performed, followed by a tracheostomy and introduction of chronic mechanical ventilation support with a Nellcor Puritan-Bennet 740 ventilator. The progression of the disease could be clearly observed over the course of the next six years. The upper and lower extremities became totally paralyzed, and the bulbar nerves became afflicted – the patient lost the ability to move, swallow, and whisper. With time, communication with the patient became possible only with the use of an alphabet table or a text editor supported by a photomechanically controlled keyboard linked to a computer. The first signs of cataracts appeared in the summer of 2005. Deterioration of eyelid winking occurred simultaneously. The rapid maturation of the cataract resulted in a nearly complete loss of communication with the patient – he was unable to use either the electronic writing device or the alphabet table. A decision regarding surgical treatment of the cataract was made by a council consisting of the engaged physicians and the consulting ophthalmologists.

The first phacoemulsification of the right eye took place on November 17, 2005. Preparation for this operation included the insertion of a central venous catheter via the left subclavian vein, and insertion of a urinary catheter. Before entering the operation room (OR), the patient received premedication consisting of pethidine (1 mg·kg^-1^) and midazolam (0.1 mg·kg^-1^) applied intramuscularly. ECG monitoring, non-invasive arterial blood pressure, pulsoximetry, and capnometry were carried out in the OR. General anaesthesia was introduced by a bolus injection of propofol (1 mg·kg^-1^), and was supported by volatile anaesthesia consisting of 65 vol% N_2_O, 0.7 vol% sevoflurane, and oxygen administered by a volume controlled anaesthesia ventilator through the tracheostomy tube, using a circle circuit with a CO_2 _absorber. Furthermore, the operating surgeon anaesthetized the eye by topical administration of proxymetacaine 0.5% eye drops. The ophthalmologic procedure lasted 25 minutes, without any complications. Following the operation, delivery of the volatile anaesthetics was ceased and the patient was transported back to the recovery room. He became responsive to alerts 20 minutes after discontinuation of the volatile anaesthetics. The total anaesthesia handling in the OR lasted 40 min, and the stay in the recovery room lasted 3 hours.

The outcome from the first operation was complicated by development of an endophthalmitis with choroids inflammation, due to a methicillin-resistant *Staphylococcus epidermidis *strain. Although the infection was successfully treated with topical and systemic antibiotics, corticosteroids, and non-steroid anti-inflammatory drugs, residual postinflammatory membranes impaired the vision and necessitated further surgical treatment. The second operation, consisting of the phacoemulsification of the left eye and a vitrectomy due to the endophthalmitis of the right eye, took place on December 20, 2005. The preparation for the operation was similar to the first one. Again, general anaesthesia was introduced by an intravenous bolus of propofol and sustained by volatile anaesthesia. For the most painful stages of the operation (vitrectomy), an additional bolus of propofol and fentanyl was administered. The ophthalmologic procedures lasted 100 min., the anaesthesiologic – 130 min. The patient stayed again for two hours in the recovery room before he was transported back to his room.

The treatment and rehabilitation after the second operation took about three months, but resulted in substantial improvement in vision and the maintenance of satisfactory communication with the patient. Two years after the ophthalmologic interventions the patient remained in a relatively stable condition, with communication still intact.

## Discussion

The progressive stage of the ALS and no publications describing the anaesthesiological procedures for ophthalmologic surgery in patients with this disease provided a serious challenge to the handling team.

Amyotrophic lateral sclerosis is a progressive, usually lethal, neurodegenerative disease that occurs mainly after the age of 50. It is the most common and most severe of the motor neuron diseases [2-4]. The annual incidence rate is roughly 1–2.5 per 100,000 [5-7]. Although hereditary cases and the familial coincidence of ALS with dementia and parkinsonism have been described, in the majority of patients the disease onset is sporadic [3,4]. The precise pathological mechanism leading to the death of motor neurons is complex and not well understood. The known molecular pathways that may be involved are more clearly identified in familial ALS [3,4]. Pathological insights are primarily derived from animal studies and include, among others, defects of superoxide dismutase – 1, disorganization of the intermediate filament, abnormal intracellular free calcium levels, and glutamate excitotoxicity which are considered to be responsible for mitochondrial abnormalities and energetic distress leading to cell injury and motor neuron death [3,4,8]. In addition, recently published genomic studies identified a few other genetic loci significantly associated with ALS [9].

ALS presents with muscle fasciculation, palsy, and spasticity, and usually results in death from respiratory failure due to hypoventilation within one to three years [4]. In some cases the onset is marked by bulbar nerve involvement, and then the disease presents with dysarthria, dysphagia, and dyspnea. Mechanical ventilation support can prolong the survival. Non-invasive methods of ventilation support are preferred before tracheostomy, but in 1999 non-invasive ventilation was not available to the handling team [10]. Therefore, invasive mechanical ventilation could only be offered to the patient as an alternative to palliative care.

At our Ophthalmology Department the phacoemulsification of a cataract is routinely done under topical anaesthesia with proxymetacaine 0.5% eye drops and/or peribulbar anaesthesia with lidocaine 2%, which is administered by the surgeon [11,12]. An anaesthesiologist usually assists the procedure with a monitored advice. The sedated, but not anaesthetized patient remains responsive to the surgeon and anaesthesiologist, allowing for the administering of additional topical or intravenous analgetics on request when regional anaesthesia causes anxiety or provides insufficient pain relief.

In the presented case the patient's alerts would be unidentifiable by the supervising anaesthesiologist due to the advanced motor paralysis and tracheostomy. Therefore, the method of choice, considered by the authors to guarantee the most comfortable state through the operation, was a combination of topical anaesthesia with slight general volatile anaesthesia. An intravenous bolus of propofol was used for a swifter induction of anaesthesia.

A special consideration must be taken into account for patients with ALS that require a neuromuscular blockade. Nondepolarising neuromuscular blocking drugs are routinely used during general anaesthesia to achieve sufficient muscle relaxation, providing good conditions for performing surgical procedures. However, an increased susceptibility to nondepolarising neuromuscular blocking drugs is present in patients with motor neuron disease. These drugs should either be entirely avoided in those patients or only used with caution following a regional curare test, under the control of a peripheral nerve stimulation monitor [12-15]. Depolarizing neuromuscular blocking drugs may also elicit myotonic reactions and massive hyperkalaemia in these patients [1,16-19]. In the presented case the authors avoided neuromuscular blocking drugs and, furthermore, there was no need for them due to the disease progression.

The arterial blood pressure and heart rate remained stable throughout both operations. In addition, no other signs of pain perception, such as sweating or rush, were noted. A follow-up on the day after the operation confirmed the patients' full satisfaction from the applied method in both operations.

## Conclusion

In preparing the algorithm for the anaesthesiological procedures used in the presented case, the authors compiled clinical experience and scarce information from literature. The applied combination of topical and volatile anaesthesia with intravenous induction was well tolerated, and provided no side-effects in a patient with advanced amyotrophic lateral sclerosis, who sustained two operations for a cataract and a vitrectomy. The applied treatment resulted in substantial vision improvement and allowed continued communication with the patient.

## Abbreviations

ALS: amyotrophic lateral sclerosis; OR: operation room.

## Consent

Written informed consent was obtained from the patient for publication of this case report. A copy of the written consent is available for review by the Editor-in-Chief of this journal.

## Competing interests

The authors declare that they have no competing interests.

## Authors' contributions

MMK, TS, and RP collected the patient's data, the consent from the patient, and partially wrote the manuscript. MMK, RS, MW, and KR were directly engaged in providing the anaesthesiological and ophthalmological procedures, as well as writing the relevant parts of the manuscript. HT and WN edited the portion of the manuscript regarding the neurological history. All authors read and approved the final manuscript.
